# Apolipoprotein A-I Attenuates Palmitate-Mediated NF-κB Activation by Reducing Toll-Like Receptor-4 Recruitment into Lipid Rafts

**DOI:** 10.1371/journal.pone.0033917

**Published:** 2012-03-30

**Authors:** Andrew M. Cheng, Priya Handa, Sanshiro Tateya, Jay Schwartz, Chongren Tang, Poulami Mitra, John F. Oram, Alan Chait, Francis Kim

**Affiliations:** Department of Medicine, Diabetes and Obesity Center of Excellence, University of Washington, Seattle, Washington, United States of America; National Institutes of Health, United States of America

## Abstract

While high-density lipoprotein (HDL) is known to protect against a wide range of inflammatory stimuli, its anti-inflammatory mechanisms are not well understood. Furthermore, HDL's protective effects against saturated dietary fats have not been previously described. In this study, we used endothelial cells to demonstrate that while palmitic acid activates NF-κB signaling, apolipoprotein A–I, (apoA-I), the major protein component of HDL, attenuates palmitate-induced NF-κB activation. Further, vascular NF-κB signaling (IL-6, MCP-1, TNF-α) and macrophage markers (CD68, CD11c) induced by 24 weeks of a diabetogenic diet containing cholesterol (DDC) is reduced in human apoA-I overexpressing transgenic C57BL/6 mice compared to age-matched WT controls. Moreover, WT mice on DDC compared to a chow diet display increased gene expression of lipid raft markers such as Caveolin-1 and Flotillin-1, and inflammatory Toll-like receptors (TLRs) (TLR2, TLR4) in the vasculature. However apoA-I transgenic mice on DDC show markedly reduced expression of these genes. Finally, we show that in endothelial cells TLR4 is recruited into lipid rafts in response to palmitate, and that apoA-I prevents palmitate-induced TLR4 trafficking into lipid rafts, thereby blocking NF-κB activation. Thus, apoA-I overexpression might be a useful therapeutic tool against vascular inflammation.

## Introduction

Low levels of high-density lipoprotein (HDL) cholesterol are associated with increased risk of coronary artery disease and major cardiovascular events. HDL-raising strategies are being evaluated for the prevention and treatment of coronary artery disease. HDL may mediate atheroprotective effects by stimulation of eNOS-dependent NO production, mediation of endothelial repair, and promotion of cholesterol efflux from macrophage foam cells [1], [2], [3], [4], [5]. In addition, HDL possesses powerful anti-inflammatory and anti-atherogenic properties by decreasing expression of cytokine-stimulated adhesion molecules, such as ICAM-1, VCAM-1 and E-selectin-1 in endothelial cells [6], [7], and attenuating expression of monocyte chemotactic protein, MCP-1 in the vasculature [8]. Since HDL is known to exert anti-inflammatory effects against a wide range of inflammatory agents such as oxidized low-density lipoproteins (LDL) [9] oxidized phospholipids [10] and 7-ketocholesterol [11], we sought to investigate whether HDL attenuates vascular inflammatory responses mediated by saturated fats such as palmitate.

Apolipoprotein A–I (apoA-I), the major protein constituent of HDL is able to recapitulate many protective functions of HDL [2], [3], [12], [13]. One mechanism by which apoA-I is believed to be anti-inflammatory is by mediating cellular cholesterol efflux through ABCA1, an ATP-binding transporter in macrophages [14], [15]. Several studies have demonstrated apoA-I to be anti-inflammatory in different animal models: *in vivo* apoA-I infusion was shown to be protective to rabbits when subjected to acute inflammation [16]. Also, apoA-I mimetic peptides, D-4F and L-4F, reduced vascular inflammation induced by streptozotocin injection in Sprague-Dawley rat [17] and improved insulin sensitivity in a mouse model of diabetes and obesity [18]. Based on these findings, we sought to study the role of HDL, and its predominant protein component, apoA-I on saturated fatty acid-induced inflammation in endothelial cells. Further, we hypothesized that apoA-I overexpressing transgenic mice would be protected from inflammatory effects of a high-fat, atherogenic diet.

Moreover our *in vitro* studies with endothelial cells suggest a mechanism by which apoA-I protein exerts the protective functions of HDL. ApoA-I prevents TLR4 migration into lipid rafts, and thereby reduces NF-κB activation in response to palmitate.

## Materials and Methods

### Animal studies

Wild type C57BL/6 and apoA-I transgenic mice were purchased from the Jackson labs. All animals were maintained in a temperature-controlled facility with a 12 hour light-dark cycle. WT (n = 7 on DDC and n = 5 on chow) and apoA-I transgenic mice (n = 7 on DDC and N = 7 on chow) of C57BL/6 background at 6–8 weeks of age were put on a diabetogenic diet containing cholesterol at 0.15% w/w (abbreviated as DDC, BioServ F4997; the diabetogenic diet provides 35.5% calories as fat and 36.6% as carbohydrate) or a standard rodent chow diet (providing 4% calories as fat) for 24 weeks [19]. At the end of the study period, the mice were sacrificed and the thoracic aortae were collected in RNAlater® (Ambion, Austin, TX) and stored at −20°C until used for RNA extraction. All experimental procedures were undertaken with approval from the Institutional Animal Care and Use Committee of the University of Washington.

### Reagents

Human ICAM-1 antibody, and Human IL-6 ELISA kit was purchased from R&D systems. HDL was prepared as previously described [20]. ApoA-I was purchased from Academy Bio-medical Company, Inc, Houston, TX. M βCD (methyl-beta-cyclodextrin) and cyclodextrin-cholesterol (CD-cholesterol) were purchased from Sigma-Aldrich. Antibodies against Caveolin-1 and phosphorylated-p65 subunit of NF-κB (used at 1∶1000 dilution) were obtained from Cell Signaling. TLR4 antibodies (used at 1∶500) and Alexa-594-conjugated Cholera-Toxin-B (CTx-B) were obtained from Invitrogen. Anti-CTx-B antibodies were obtained from Calbiochem. Antibodies against GAPDH (used at 1∶2000) was obtained from Santa Cruz Biotechnology. Palmitic acid (C16∶0) fatty acids were obtained from Alltech Associates Inc., and BSA (bovine serum albumin, free-fatty acids (FFA)-free) was purchased from Roche. FFA were dissolved in 0.1 mol/L NaOH at 70°C and then complexed with 10% BSA at 55°C for 10 minutes. Stock solutions of 5 mmol/L FFA with 10% BSA and 10% BSA were prepared a day before the experiments and diluted in endothelial cell culture media to achieve a final palmitate concentration of 100 µmol/L as described previously [21]. Optiprep (60% Iodixanol) was purchased from Sigma-Aldrich and used to generate a step gradient consisting of 5%, 30% and 40%.

#### Cell culture

Human microvascular endothelial cells (HMEC) were purchased from Invitrogen-Cascade Biological and were cultured as previously described [21]. Bovine aortic endothelial cells (BAEC) were purchased from Clonetics and cultured as previously [22]. IL-6 ELISA was performed as per the instructions of the manufacturers. Western blotting was performed with equal amounts of total protein for each condition and experiment as described [21].

#### Isolation of lipid rafts using Optiprep gradient centrifugation

Lipid raft isolation was performed as follows: BAEC or HMEC were grown to confluence in 10 cm dishes. They were treated with the various agents (palmitic acid, with or without pretreatment with MβCD or apoA-I in 10% lipid-deficient serum in DMEM) for the designated durations and doses. They were then washed, the cells trypsinized, washed twice with phosphate buffered saline (PBS), resuspended in 133 µl of cell lysis buffer at 4°C. Lysis buffer comprises of 1% Triton-X-100, 25 mM Tris HCl, pH 7.4, 5 mM EDTA, 1 µM sodium orthovanadate, 100 µM DTT, 200 µM PMSF, 10 µg/ml and protease inhibitors. The cells were sonicated to ensure lysis. Next, 133 µl of the lysate was mixed with 267 µl of the 60% optimix solution (Sigma) to form the bottom most layer of the gradient in the ultracentrifuge-compatible microfuge tube. The next layer was generated using 700 µl of the 30% optimix solution and the final layer was composed of a 5% optimix solution. The microfuge tubes were spun in a tabletop ultracentrifuge for 18 hours at 100,000 g at 4°C in a TLA-55 bucket in a Beckman rotor. The gradient was divided into 10 equal parts and fractions 2-5 represented the lipid raft fraction and the fractions 6–9 represented the non-lipid raft fraction. Protein concentration was determined using Pierce BCA protein assay (Piece, Rockford, IL). The pooled fractions were resuspended in sample lysis buffer boiled for 5 minutes and stored in −20°C till analyzed by western blotting.

#### Western blotting

SDS gel electrophoresis was performed using a 4% by 20% gradient gel. Quantification of Western blots was performed using Image J Processing and Analysis (NIH).

#### Quantitative RT-PCR Analyses

RNA was extracted using RNAeasy kit (Qiagen). For gene expression analysis, real-time-PCR reactions were performed using TaqMan Gene Expression Analysis from Applied Biosystems as described previously [21] and normalized to GAPDH levels. Human ICAM-I, or the Mouse IL-6, MCP-1, TNF-α, CD68, CD11c, Caveolin-1, Flotillin-1, TLR4 and TLR2 RT-PCR primer pairs were purchased from Applied Biosystems.

#### Cholesterol efflux measurement

BAEC were labeled with 1 µCi/ml of ^3^[H] cholesterol (PerkinElmer Life Sciences) for 24 hours. The cells were washed and then incubated with or without human apoA-I (10 µg/ml) for 8 hours in DMEM/BSA. Cholesterol efflux was measured by counting ^3^[H] in the medium and the cell extracts. ApoA-I-mediated cholesterol efflux was calculated as the percent total [^3^H]cholesterol released into medium after subtraction of values obtained in the absence of apoA-1 [14].

#### Immunofluorescent microscopy of lipid rafts

Endothelial cells were grown on coverslips within a 6-well plate at the density of 200,000/well. After overnight growth, they were treated with either HDL (50 µg/ml) or apoA-I (50 µg/ml) for 16 hours or M βCD (1 mM for 30 mins), or CD-cholesterol (40 µg/ml for 1 hour). After the respective treatment times, they were washed multiple times before being treated with Alexa-594 conjugated to Cholera-toxin-B (CTx-B, 1 µg/ml) at 4°C for 10 minutes in the dark. They were washed 3 times and incubated with anti-CTx-B antibodies (4 µg/ml, Calbiochem) for 15 mins, washed with PBS thrice, and treated with DAPI (1 ng/ml), followed by washing with PBS 3 more times. Subsequently, cells were fixed in 4% paraformaldehyde in PBS for 15 minutes. The coverslips with the cells were then placed on microscopic glass slides, which were treated with Biomeda's aqueous mounting medium containing anti-fading solution. For imaging, an Olympus fluorescence microscope was used and images collected using appropriate filter sets. The quantification of the fluorescent intensities was done by the NIS-elements software. The fluorescent intensities of about 30-50 cells were measured in each experiment, and expressed as arbitrary fluorescence units.

#### Statistical analysis

All results are represented as mean ± SEM. Results were analyzed using a 2-tailed Student's *t-* test or one- or two-way ANOVA, where appropriate using Graphpad Prism software. A Bonferroni post hoc analysis was done to compare mean values between groups. P<0.05 was considered to be significant and marked *, P<0.01 was marked **, p<0.001 was marked ***.

## Results

### Diabetogenic diet-induced vascular inflammation is reduced in apoA-I transgenic mice

**Figure 1:**
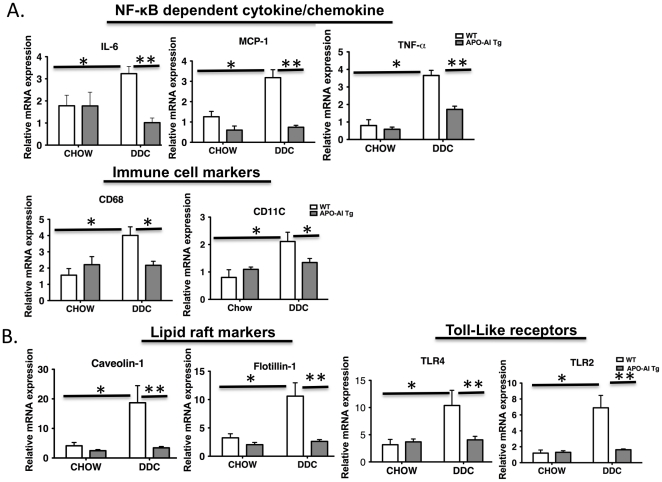
ApoA-I overexpression attenuates the effect of DDC on vascular inflammation and lipid raft markers. C57BL/6 (WT) or apoA-I transgenic mice were fed a standard (chow) or a DDC diet for 24 weeks and gene expression from isolated thoracic aortic tissues were assessed by quantitative RT-PCR. **A**. Fold differences between groups were calculated for MCP-1, IL-6 and TNF-α (NF-κB dependent signaling) CD68, CD11c (monocyte markers) relative to GAPDH. **B.** Fold differences between groups were calculated for Caveolin-1 and Flotillin-1 (lipid raft markers), TLR2 and TLR4 (Toll-like receptors) relative to GAPDH. *P<0.05, **p<0.001, n = 4–7 for each condition.

We first asked whether overexpression of apoA-I, an integral component of HDL, would reduce high-fat, high-sucrose diet-dependent vascular inflammation [23]. WT C57BL/6 and apoA-I transgenic mice were maintained on a normal chow diet or a diabetogenic diet (DD) plus 0.15% cholesterol containing diet (DDC) for 24 weeks. At the end of the study period, mice were sacrificed, thoracic aortae were carefully removed and cleaned of adhering adipose tissue. As expected, gene expression of NF- κB dependent signaling (IL-6, MCP-1, TNF-α) and monocyte/macrophage/dendritic cell markers (CD68 and CD11c) increased in response to 24 weeks of DDC, whereas, overexpression of human apoA-I *in vivo* attenuated these responses (**Figure 1A**).

Lipid rafts, cholesterol-rich microdomains in the plasma membranes, serve as recruiting platforms for many immune receptors in response to their cognate ligands involved in inflammatory signaling (eg. TLR4, TNF-receptor) [24], [25]. Perturbation of lipid raft composition or altering the number of rafts is known to affect inflammatory signaling [24], [26], so we next asked whether apoA-I overexpression is associated with alterations in lipid rafts. The DDC diet increased lipid raft markers (caveolin-1, flotillin-1), a response not seen in apoA-I transgenic mice (**Figure 1B**). Thus apoA-I overexpression is associated with reduced lipid raft markers.

TLR2 and TLR4 are recruited into lipid rafts in response to lipoproteins and LPS respectively to cause NF-κB activation [24], [26], [27]. Thus, we assessed the gene expression levels of the TLR2 and −4 in our diet-induced obesity (DIO) studies and found that while DDC significantly elevated the levels of expression of these inflammatory mediators, the apoA-I transgenic animals had markedly reduced expression (**Figure 1B**). These data collectively suggest that apoA-I overexpression is protective towards vascular inflammation by reducing NF-κB mediated inflammatory mediators such as IL-6, MCP-1, TNF-α, caveolin-1, flotillin-1, TLR2 and TLR4. ApoA-I overexpression also reduced immune cell markers, CD68 and CD11c expression, presumably due to reduced expression of recruiting chemokines such as MCP-1. We did not see significant changes in M2 markers such as IL-10 and Arginase-1 (data not shown).

### Palmitate induced NF-κB activation is attenuated by HDL or apoA-I in endothelial cells *in vitro*

**Figure 2:**
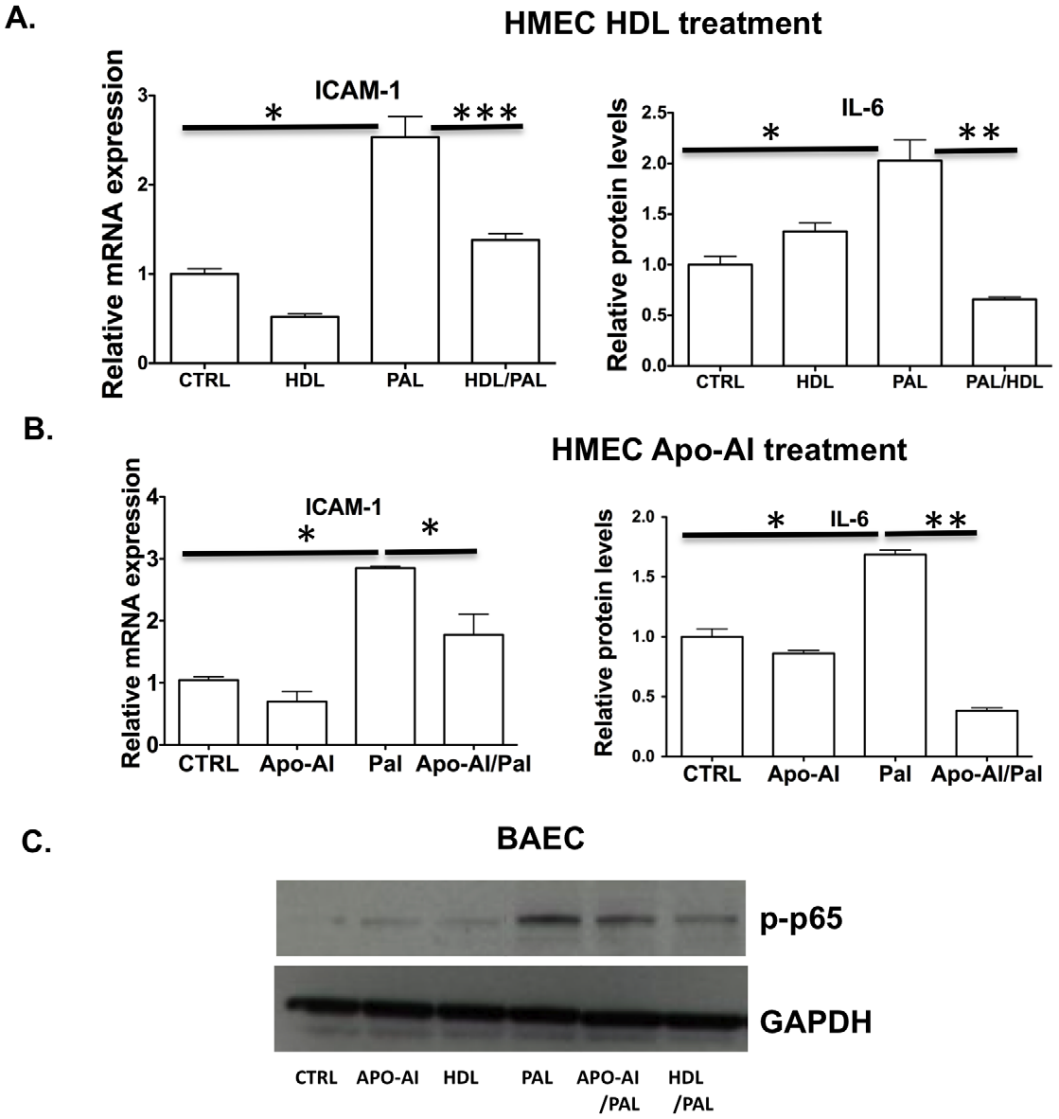
HDL or apoA-I attenuates palmitic acid dependent NF- κB signaling in endothelial cells. HMEC were pretreated with vehicle (labeled as control) or with human HDL (50 µg/ml) or ApoA-1 (50 µg/ml) for 16 hours, washed and then either treated with palmitate complexed with BSA (100 µM) for 3 hours or treated with BSA alone. **A–B**. ICAM-1 mRNA expression was analyzed using quantitative RT-PCR. IL-6 cytokine levels in supernatants were assessed by ELISA (n = 3). Data represents mean ± SD and *p<0.05,**p<0.01,***p<0.005. **C**. BAEC lysates were prepared after pretreatment with apoA-I/HDL and phopho-P65 levels were assessed by Western blot.

HDL/apoA-I reduces NF-κB activation mediated by many stimuli such as LPS, TNF-α, and oxidized LDL [6], [7], [13], [28]. We next asked whether HDL/apoA-I would similarly reduce palmitate-dependent activation of endothelial NF-κB. Human microvascular endothelial cells (HMECs) were pretreated with 50 µg/ml of HDL or apoA-I (50 µg/ml) for 16 hours, washed and subsequently treated them with 100 µM of palmitate for 3 hours. Pretreatment with either HDL or apoA-I significantly reduced palmitate-dependent increases in the expression of ICAM-1, and IL-6 cytokine levels compared to the vehicle-treated cells (**Figure 2A, B**). These observations were also found in a model of large vessel endothelial cells (BAEC). Pretreatment with apoA-I or HDL attenuated palmitate-mediated NF-κB activation, as assessed by phosphorylation of the p65 subunit of NF-κB (**Figure 2C**). Therefore, HDL/apoA-I is able to attenuate palmitate-mediated NF-κB-dependent inflammatory responses in endothelial cells.

### Palmitate induces TLR4 migration into lipid rafts

**Figure 3:**
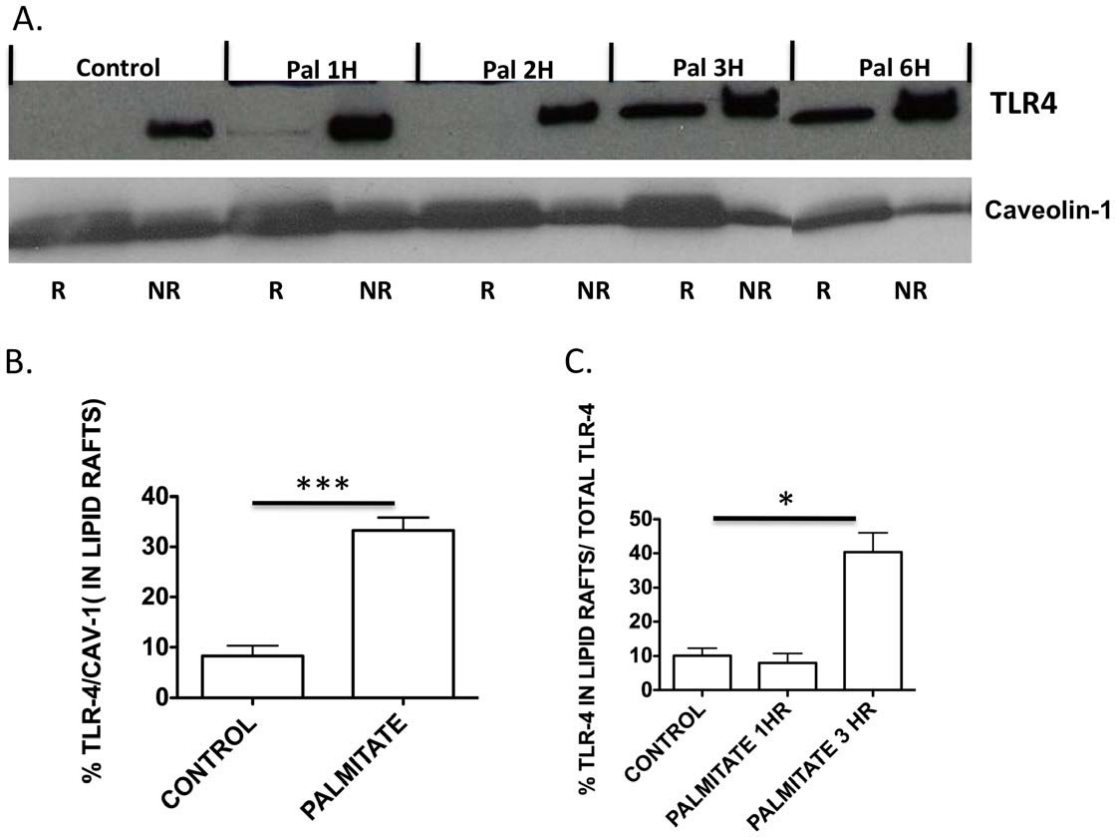
TLR4 is recruited into lipid rafts in response to palmitate. **A.** BAEC were treated with BSA alone (control) or palmitate/BSA (100 µM) for 1, 2, 3 and 6 hours. Lipid rafts were isolated by Optiprep gradient centrifugation and the raft(depicted as ‘R’) and non-raft fractions(depicted as ‘NR’) were pooled. Cell lysates were assessed for TLR4 and caveolin-1 protein levels by Western blot. Representative immunoblot from 3 independent experiments is shown. B. Densitometric quantitations of western blots comparing the band intensities of TLR4 in the raft fraction to caveolin-1 in the same raft fraction(n = 5). C. Densitometric quantitations of Western blots comparing the band intensities of TLR4 in the raft fraction to total TLR4 (lipid raft and non-raft fraction) are shown.

It was shown previously that LPS-induced TLR4 activation is initiated by recruitment of TLR4 into lipid rafts, a critical and early step in the inflammatory signaling process [24], [25]. Since TLR4 is necessary for palmitate-mediated-activation of NF-κB [21], we next examined whether palmitate increases recruitment of TLR4 into lipid rafts. HMEC were treated with palmitate (100 µM) for 1, 2, 3, and 6 hours and the raft and non-raft fractions were isolated using Optiprep gradient centrifugation. Compared to BSA alone treated HMEC (control), palmitate-BSA increases TLR4 migration into isolated lipid rafts by 3–6 hours (**Figure 3A, B**). As expected, the isolated lipid raft fractions were enriched with the caveolin-1 protein compared to the non-raft fractions (**Figure 3A**). The 3 hour time-point for TLR4 migration corresponds to the maximal NF-κB activation time-point as seen previously [21], [23]. Palmitate similarly recruited TLR4 into lipid rafts in BAEC (data not shown).

### Lipid raft integrity is important for palmitate-induced TLR4 mediated NF-κB activation

**Figure 4:**
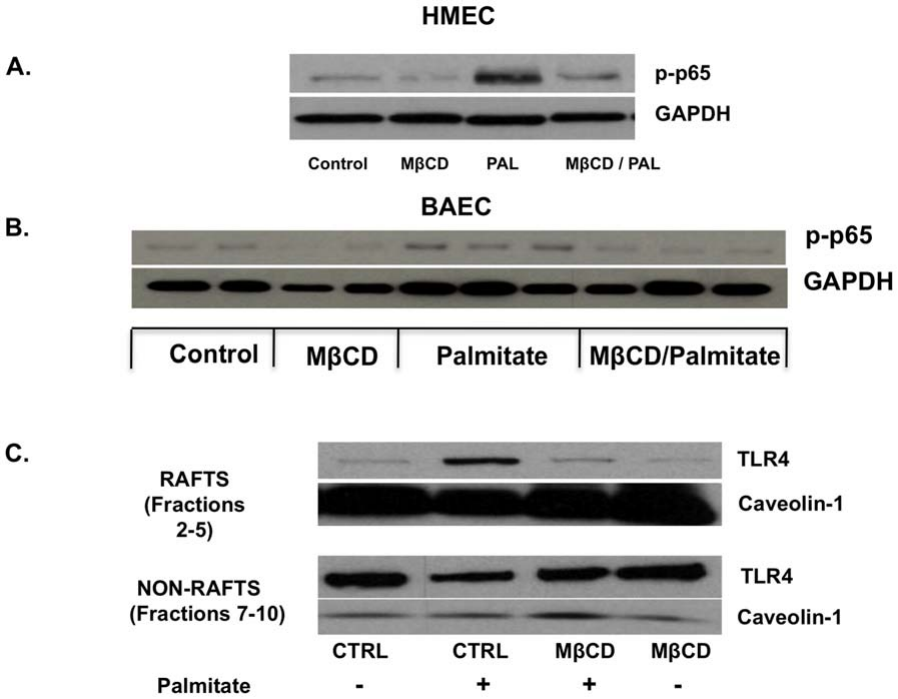
Disruption of lipid raft integrity by MβCD reduces palmitate-mediated NF- κB signaling and TLR4 migration in endothelial cells. HMEC cells were pretreated with vehicle or MβCD (5 mM/1 hr) (to disrupt lipid raft integrity) and then stimulated with palmitate/BSA (100 µM) for 3 hr. Cell lysates were assessed by Western blot with an anti-phospho-p65 antibody (n = 3). **B**. Experiments were repeated in BAEC (n = 3). **C**. Lipid rafts were isolated and the non-raft and lipid-raft fractions pooled, and assessed for TLR4 and caveolin-1 protein level by Western blot. Representative immunoblots from 3 independent experiments are shown.

We next asked whether the structural integrity of the lipid rafts is required for palmitate-induced TLR4-mediated endothelial cell activation. Methyl-beta-cyclodextrin (MβCD), a cholesterol-effluxing agent, disrupts the integrity of the lipid rafts by reducing the cholesterol content in the lipid rafts [24]. HMEC/BAEC were pretreated with vehicle or exposed to 5 mM MβCD for 1 hour prior to exposure to palmitate (100 µM) for 3 hours. Pretreatment with MβCD reduced palmitate-mediated phosphorylation of p65 subunit (**Figure 4A, B**) and reduced the recruitment of TLR4 into the lipid rafts (**Figure 4C**). Thus, functional lipid rafts are required for the palmitate induced TLR4 mediated NF-κB activation in endothelial cells.

### ApoA-I reduces lipid raft abundance and reduces TLR4 migration into lipid rafts

**Figure 5:**
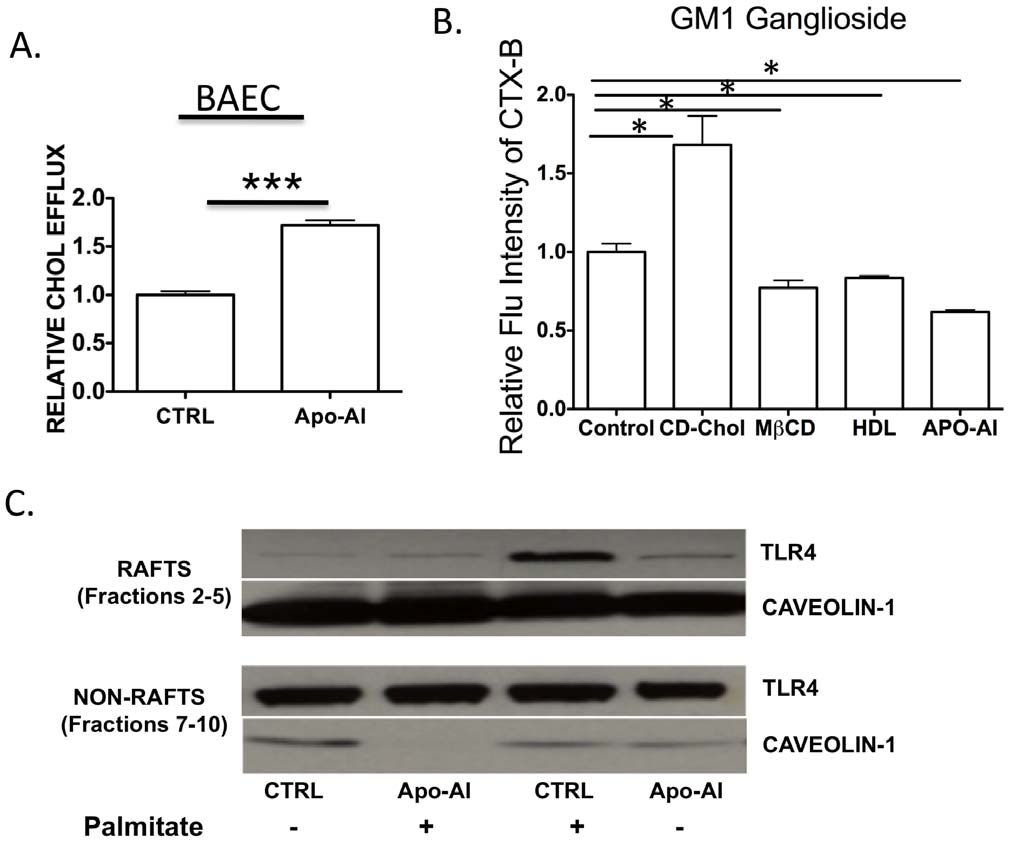
Apo-AI increases endothelial cholesterol efflux, reduces lipid raft content and TLR4 migration into lipid rafts. **A**. BAECs were labeled with 1 µCi/ml of ^3^[H] cholesterol (PerkinElmer Life Sciences) for 24 hours. The cells were washed and then incubated with vehicle or human ApoA-1 (10 µg/ml) for 8 hours in DMEM/BSA. Cholesterol efflux was measured by counting ^3^[H] in the medium and the cell extracts. ApoA-I-mediated cholesterol efflux was calculated as the percent total [^3^H]cholesterol released into medium after subtraction of values obtained in the absence of apoA-I (n = 3–7), ***p<0.001 indicates significance. **B.** Lipid raft staining was done and visualized by fluorescence microscopy, using fluorescent cholera-toxin-B, which binds specifically to GM1 ganglioside. Positive control (increase cholesterol content: CD-cholesterol) and negative control (reduce cholesterol content: (MβCD)) were used. Endothelial cells were treated with either HDL or apoA-I (50 µg/ml) for 16 hours, MβCD (1 mM) or CD-cholesterol (20 µg/ml). The data has been represented as relative fluorescent intensities compared to untreated cells. In each experiment, 30–50 cells were counted (n = 2). **C.** BAEC were pretreated with apoA-I for 16 hours prior to treating with palmitic acid for three hours. The cells were then washed with PBS, trypsinized, lipid raft and non-raft fractions isolated. The pooled raft and non-raft fractions were assessed for TLR4 and Caveolin-1 proteins by Western blot. Representative immunoblot from 4 independent experiments is shown(n = 4).

ApoA-I is known to cause cholesterol efflux from macrophages [14], [15]. Previously, Murphy and coworkers showed that HDL/apoA-I reduces lipid raft content in monocytes and neutrophils as assessed by GM1 ganglioside staining, a component of lipid rafts [29], [30]–[31]. We next asked whether apoA-I exerts its anti-inflammatory effect in endothelial cells via cholesterol removal, thereby disrupting lipid raft signaling. Treatment with purified human apoA-1 increased cholesterol levels in the media, suggesting that apoA-1 enhances cholesterol endothelial efflux (**Figure 5A**). Next we asked whether apoA-I induced cholesterol efflux in endothelial cells is associated with a reduction in lipid raft content. We tested this hypothesis by direct visualization of total lipid rafts by fluorescence microscopy using labeled cholera toxin-B (CTx-B), which binds specifically to GM1 ganglioside, a lipid raft marker. We found that cholesterol- depleting agents such as MβCD, apoA-I or HDL treated endothelial cells had lower lipid raft abundance relative to untreated endothelial cells. In contrast, cholesterol-loaded cells (by CD-cholesterol) have higher lipid raft content (**Figure 5B**), which was previously shown to be associated with enhanced NF-κB activation [30]. Collectively these data suggest that apoA-I exerts an indirect anti-inflammatory effect by virtue of reducing the lipid raft content by cholesterol removal.

We next pretreated BAEC with apoA-I (100 µg/ml) for 16 hours in lipid-free serum containing media, before treating with palmitate (100 µM) for three hours. Pooled lipid raft fractions were assessed for TLR4 and caveolin-1 protein levels by Western blot analysis. Palmitate induced TLR4 recruitment was abrogated by apoA-I pretreatment (**Figure 5C**). Thus, apoA1 reduces palmitate-mediated TLR4 recruitment into lipid rafts by increasing cholesterol efflux and disrupting lipid rafts. This finding is consistent with studies that showed human apoA-I [32] or apoA-I mimetic peptide, 4F [33] reduces TLR4 expression on the cell surface of endothelial cells and monocytes.

## Discussion

In the setting of diabetes and obesity, elevated free fatty acids are associated with the pathogenesis of inflammation in the vasculature and peripheral tissues. At a molecular level, free fatty acids activate IKK-κ/NF-κB pathway via TLR4 leading to increased expression of adhesion molecules such as ICAM-1 and VCAM-1, and release of proinflammatory cytokines such as IL-6 and TNF-α. While, cholesterol and dietary saturated fats increase vascular inflammation, HDL and its constituent protein, apoA-I have been shown to reduce inflammatory signaling [1], [4], [13]. In the present study, we examined the ability of HDL or apoA-I, to reduce these vascular inflammatory responses. We found that, both, HDL or apoA-I, attenuates palmitate-dependent NF-κB activation in endothelial cells *in vitro* by reducing TLR4 migration into lipid rafts. Similarly, we found that unlike its WT counterparts, apoA-I transgenic mice were protected from diabetogenic diet-induced vascular inflammation.

Our study suggests a possible mechanism by which apoA-I exerts its anti-inflammatory effects through modification of lipid rafts and secondary effects on TLR4 and NF-κB signaling. Caveolin-1 is a fundamental structural protein of the caveolae/lipid raft membrane domains [34], [35]. It is known to bind to cholesterol, interact with and negatively regulate endothelial nitric oxide synthase (eNOS) and stimulate the expression of proatherogenic molecules such as CD36, a fat transporter, and VCAM-1 [34]. Caveolin-1 levels have been shown to be elevated in the aorta of rabbits fed a high-fat diet [36] and is elevated in response to endotoxin via an NF-κB-dependent pathway in endothelial cells [37], [38]. Ablation of endothelial caveolin-1 protects ApoE−/− mice (a mouse model of atherosclerosis), from developing atherosclerosis [34], [35]. Consistent with these observations, our study shows that WT mice fed a high-fat diet have higher levels of Caveolin-1 expression and markedly increased vascular inflammation compared to chow-fed WT mice. However in the human apoA-I transgenic mice fed a high-fat diet, Caveolin-I expression is significantly reduced with a parallel reduction in vascular inflammation. There is also a similar reduction in Flotillin-1 expression, another critical structural component of the planar lipid rafts [26]. Together these observations suggest that lipid rafts may play a fundamental role in high-fat induced vascular inflammation, and that apoA-I may affect diet-induced inflammation by either directly or indirectly altering lipid rafts. Our observation that apoA-I effluxes cholesterol from the plasma membranes of BAEC further supports this model since cholesterol depletion can lead to lipid raft disruption and hence reduced inflammatory signaling.

Our *in vivo* data also show that apoA-I is associated with decreased TLR2 and −4 expression. It has been well-established that expression of TLRs play an important role in innate immunity but also in mediating NF-κB-induced-inflammation in DIO models. Previously, Tall and coworkers showed that macrophages of mice lacking ABC transporters have elevated surface expression of TLRs, specifically TLR4/MD-2, associated with increased inflammation as compared to macrophages from WT mice [30]. In rodent DIO models, TLR2 and −4 expression levels were found to be augmented in adipose tissue [39], and vasculature [40]. These findings support our finding that TLR2 and −4 expression were up-regulated in DDC-fed WT mice while the apoA-I transgenic mice on DDC were protected from this effect.

In this study we have further established an interaction between TLR4 and lipid rafts in a high-fat induced inflammatory model. We previously showed that the mechanism by which palmitate causes endothelial dysfunction involves TLR4-mediated induction of the inflammatory NF-κB pathway in endothelial cells [21]. In this report, we extend our observation by showing that in endothelial cells TLR4 is recruited into lipid rafts in response to palmitate. Our finding that saturated fat exemplified by palmitate causes TLR4 recruitment into lipid rafts to induce inflammatory signaling is consistent with a earlier study by Hwang and coworkers, who demonstrated this phenomenon in macrophages [41].

We further demonstrate that apoA-I disrupts the interaction between TLR4 and lipid rafts by impairing recruitment of TLR4 into the rafts. Since apoA-I is known to cause cholesterol efflux out of cell membranes, we sought cholesterol acceptors also known to remove cholesterol from cells to see if they would have similar effects as apoA-I. Indeed, MβCD reduced palmitate-induced TLR4 recruitment into lipid rafts and NF-κB activation suggesting that palmitate induced NF-κB activation requires functional lipid rafts and cholesterol efflux can inhibit this process.

Finally we looked downstream of TLR4 signaling and studied apoA-I's effects on NF-κB activation. TLR4 activates NF-κB induced inflammation in endothelial cells. We show that apo-AI addition to endothelial cells prevents TLR4-mediated NF-κB activation. Our study is consistent with the other studies, which show that treatment of human monocytes with apoA-I or apoA-I mimetic 4F leads to reduced surface expression of TLR4 and CD11c [33]. Another study found that there is reduction in chemokines such as MCP-1, and chemokine receptors such as CCR2 in atherosclerotic plaques of ApoE−/− mice in response to apoA-I injections [42].

In conclusion, apo-AI overexpression attenuates high fat mediated-vascular inflammation by reducing the expression of chemokines and cytokines in vivo. Similarly, in endothelial cells, HDL/apo-AI attenuates palmitate-induced NF-κB activation by scavenging cholesterol from plasma membrane domains, thereby reducing total lipid raft content, decreasing TLR4 migration into lipid rafts resulting in reduced TLR4 signaling.
